# Scientific Justification of Cryonics Practice

**DOI:** 10.1089/rej.2008.0661

**Published:** 2008-04-01

**Authors:** Benjamin P. Best

**Affiliations:** Cryonics Institute, Clinton Township, Michigan.

## Abstract

Very low temperatures create conditions that can preserve tissue for centuries, possibly including the neurological basis of the human mind. Through a process called vitrification, brain tissue can be cooled to cryogenic temperatures without ice formation. Damage associated with this process is theoretically reversible in the same sense that rejuvenation is theoretically possible by specific foreseeable technology. Injury to the brain due to stopped blood flow is now known to result from a complex series of processes that take much longer to run to completion than the 6 min limit of ordinary resuscitation technology. Reperfusion beyond the 6 min limit primarily damages blood vessels rather than brain tissue. Apoptosis of neurons takes many hours. This creates a window of opportunity between legal death and irretrievable loss of life for human and animal subjects for cryopreservation with possibility of future resuscitation. Under ideal conditions, the time interval between onset of clinical death and beginning of cryonics procedures can be reduced to less than 1 min, but much longer delays could also be compatible with ultimate survival. Although the evidence that cryonics may work is indirect, the application of indirect evidence is essential in many areas of science. If complex changes due to aging are reversible at some future date, then similarly complex changes due to stopped blood flow and cryopreservation may also be reversible, with life-saving results for anyone with medical needs that exceed current capabilities.

## Introduction

Cryonics is the practice of preserving humans and animals at cryogenic temperatures in the hope that future science can restore them to a healthy living condition as well as rejuvenate them. At present cryonics can only be performed after pronouncement of legal death of the cryonics subject.

The scientific justification for the practice of cryonics is based on several key concepts: (1) low temperature can slow metabolism. Sufficiently low temperatures can virtually stop chemical changes for centuries; (2) ice formation can be reduced or even eliminated by the use of vitrification mixtures; (3) legally dead does not mean “irreversibly dead.” Death is a process, not an event, and the process takes longer than is commonly believed; (4) damage associated with low temperature preservation and clinical death that is not reversible today is theoretically reversible in the future.

Pronouncement of legal death is necessary before cryonics procedures can begin because cryonics is not yet a proven, recognized medical procedure. Following legal death, a cryonics team can begin preservation procedures immediately. Cryonics preservation procedures are intended to protect the tissues of cryonics subjects while cooling them to temperatures below −120°C with minimal alteration of tissue structure after cardiac arrest.

In the first stage of cryopreservation, the circulation and respiration of the cryonics subject is mechanically restored, and the subject is administered protective medicines and is rapidly cooled to a temperature between 10°C and 0°C. The subject's blood is washed out and a significant amount of body water is replaced with a cryoprotectant mixture to prevent ice formation. The subject is cooled to a temperature below −120°C and held in cryostasis. When and if future medicine has the capability, the subject will be re-warmed, the cryoprotectant will be removed, tissues will be repaired, diseases will be cured, and the subject will be rejuvenated (if required).

## Cooling

Preservation of food in refrigerators and freezers is based on the principle of lowering temperature to reduce the rate of biochemical degradation. Cooling to reduce metabolic rate (and ultimately to bring chemical processes to a virtual halt) is at the heart of cryonics practice. Initial cooling after pronouncement of death involves placing the cryonics subject in a bath of ice water. Cardiopulmonary support with mechanical active compression/decompression also speeds cooling because of heat transfer from flowing blood.

Cryonics subjects are cooled with convection, a combination of conduction and fluid motion. In convection, a solid object (such as the cryonics subject) is cooled by a fluid (liquid or gas) that is rapidly circulated, such that the fluid can carry heat away from the conduction layer around the solid object. In cooling a cryonics subject from human body temperature (37°C) to 10°C, cooling by rapid circulation of ice water is far more effective than cooling by ice packs or by standing water because of the convection effect.

The formula governing convection is Newton's law of cooling, which equates the rate of heat transfer to hA(T_s_ – T_f_), where T_s_ is the starting temperature (from head or body of a cryonics subject), T_f_ is the final temperature (temperature of the cooling medium, e.g., ice water or cold nitrogen gas), A is the surface area of the solid, and h is a variable dependent on the rate of fluid motion, as well as the thermal conductivity and heat capacity of the cooling medium. Faster fluid motion and higher thermal conductivity will increase the value of h. Newton's law of cooling predicts that the cooling rate is greatest at the start of cooling when T_s_ is much greater than T_f_. The cooling rate declines exponentially thereafter.

Reduction in temperature can considerably extend the time without blood flow before irreversible damage occurs. Many people, especially children, have been reported to survive from 20 min to 1 h or more of cardiac arrest with complete neurological recovery after hypothermic accidents, such as drowning in cold water.^1,2^ Metabolic rate can be dramatically reduced by cooling.

Duration of ischemic time necessary to cause 50% neuronal damage in gerbils has been shown to increase exponentially with lowering of brain temperature from 37°C to 31°C.^3^ Six out of six experimental hypothermic dogs having tympanic temperatures of 10°C were shown to endure 90 min of cardiac arrest without subsequent neurological damage, and two out of seven endured 120 min without evident neurological damage.^4^ Humans have been subjected to deep hypothermic cardiac arrest for aortic surgery for more than 1 h without gross neurological deficits. The subjects reached complete electrocerebral silence (zero electroencephalographic bispectral index) between temperatures of 16°C and 24°C,^5^ and were rewarmed without neurological deficit, confirming that dynamic brain activity can be lost and regained without loss of personal identity.

The extension of hypothermic protection from ischemic injury to subzero temperatures is seen in the northern wood frog (*Rana sylvatica*), which can survive in a semi-frozen state without heartbeat for months at temperatures as low as −3°C to −6°C with full recovery upon re-warming.^6^ In 1966 a Japanese researcher replaced blood with glycerol to reduce ice formation in cat brains cooled to −20°C. After 45 days with no blood circulation at −20°C, the revived cat brains demonstrated normal-looking EEG activity.^7^

The relationship between reaction rate (k) of chemical reactions (including metabolism and the processes of ischemic injury) and temperature (T) can be described by the Arrhenius equation^8^:

\begin{align*}{ \rm k} = { \rm Ae}^{ - { \rm E_a} / { \rm RT}}\end{align*}

where T is in kelvins (K), E_a_ is the activation energy, R is the universal gas constant (8.314 joules (J)/mole-kelvin), and A is the frequency factor (related to frequency of molecular collisions and the probability that collisions are favorably oriented for reaction). Taking the natural logarithm of both sides of this equation gives:

\begin{align*}{ \rm ln \ k} = (  -  { \rm E_a} / { \rm RT} ) + { \rm ln \ A}\end{align*}

For each of two different temperatures, T_1_ and T_2,_ there will be a different temperature-dependent reaction rate, k_1_ and k_2_:

\begin{align*}{ \rm ln \ k}_1 = (  -  { \rm E_a} / { \rm RT}_1 ) + { \rm ln \ A}\end{align*}

\begin{align*}{ \rm ln \ k}_2 = (  -  { \rm E_a} / { \rm RT}_2 ) + { \rm ln \ A}\end{align*}

Subtracting ln k_2_ from ln k_1_ gives a single equation for the four variables:

\begin{align*}{ \rm ln \ k}_1 - { \rm ln \ k}_2 = ( (  -  { \rm E}_{ \rm a} / { \rm RT}_1 ) + { \rm ln \ A} ) - ( (  -  { \rm E_a} / { \rm RT}_2 ) + { \rm ln \ A} )\end{align*}

which can be simplified to:

\begin{align*}{\rm In} ( {\rm k}_1 / { \rm k}_2 ) = {\rm E_a} / {\rm R} )^* ( 1 / {\rm T}_2  -  1 / {\rm T}_1 )\end{align*}

or

\begin{align*}{ \rm k}_1 / { \rm k}_2 = { \rm e}^{ ( { \rm Ea} / { \rm R} ) ^* ( 1 / { \rm T}2 - 1 / { \rm T}1 ) }\end{align*}

The reaction rates of enzymes at various temperatures give a close approximation to the relationship between temperature and metabolic rate. Lactate dehydrogenase from rabbit muscle, which has an activation energy (E_a_) of 13,100 calories/mol,^9^ can be taken as a representative enzyme. Using one thermochemical calorie equal to 4.184 J gives 54,810 J/mol.

Comparing the reaction rate (k_1_) for lactate dehydrogenase at 40°C (313 K) (T_1_) to the reaction rate (k_2_) at 30°C (303 K) (T_2_) gives:

\begin{align*}{ \rm k}_1 / { \rm k}_2 = { \rm e}^{ ( ( 54 , 810 \ { \rm J} / { \rm mol} ) / ( 8.314 \ { \rm J} / { \rm mol  -  K} ) ) ^* ( 1 / 303 \ { \rm K}  -  1 / 313 \ { \rm K} ) } = 2.004\end{align*}

The reaction rate at 40°C is almost exactly twice the reaction rate at 30°C or, conversely, dropping the temperature 10°C has the effect of cutting the reaction rate nearly in half. This is in agreement with the Q_10_ rule, that is, a rule of thumb that between 0°C and 40°C reaction rates are reduced by one half to one third for every 10°C drop in temperature.^10^

This exponential drop in reaction rates with declining temperature means that reaction rates would become infinitesimally small at cryogenic temperatures (temperatures below −100°C) if chemical reactions were possible at those temperatures. Table 1 compares the reaction rate at 37°C (310 K, normal human body temperature) to reaction rates at lower temperatures. The results were produced by using the above equation.

**Table 1.** Reaction Rate at 37°C Compared to Lower Temperatures

*Temperature*	*Reference*	*Relative rate at 37°C*	*Relative to 6 min at 37°C*
0°C (273 K)	Melting ice	18	1.8 h
−80°C (193 K)	Dry ice	400,000	4.5 yr
−120°C (153 K)	Glass transition	3 billion (3 × 10^9^)	34,000 yr
−196°C (77 K)	Boiling nitrogen	9 octillion (9 × 10^27^)	100 sextillion (10^23^) yr

If lactate dehydrogenase reaction rate was representative of metabolism in general, the metabolism at 37°C would be 18 times faster than at 0°C. Experimentally it has been observed that the rate of oxidative phosphorylation at 4°C is about one-twentieth the rate at 37°C,^11^ a figure roughly in agreement with the value just calculated.

A reaction rate that is 9 octillion times faster at human body temperature than at −196°C would indicate essentially no reaction for millennia at the lower temperature. At this rate it would take 100 sextillion years for the ischemic biochemical reactions that occur at 37°C in 6 min to occur at liquid nitrogen temperature. But even these figures understate chemical inertness at lower cryogenic temperatures because the Arrhenius equation is based on the assumption of a fluid or gas medium in which normal chemistry is possible. Below −130°C even vitrified mammalian tissues are in a solid state, with a viscosity in excess of 10^13^ poise,^12,13^ a viscosity about 10^15^ (one quadrillion) times greater than the viscosity of water at 20°C.^14^ The resulting diffusion rates are insignificant over geological time spans. At liquid nitrogen temperature, mammalian tissues would even be stable against background radiation over periods of many centuries.^12^

It is a misconception that freezing mammalian tissue typically results in ice formation within cells, causing the cells to burst. As mammalian tissues are cooled, water leaves cells osmotically to form extracellular pure water-ice crystals. The unfrozen solution will contain increasing concentrations of toxic electrolytes. Ultimately enough extracellular ice will form to crush cells in the remaining unfrozen channels.^12^ Whether mechanical crushing or toxic electrolytes is the cause of damage following ice formation during slow cooling remains a subject of debate among cryobiologists.^15^ Cryonics practice, however, is based on efforts to reduce or eliminate freezing.

## Vitrification and Cryogenic Storage

Cryonics practice has long sought to minimize ice formation by perfusing cryonics subjects with anti-freeze compounds known as cryoprotectants, traditionally glycerol. As of 2007 both of the major cryonics organizations doing cryoprotectant perfusions (the Alcor Life Extension Foundation and the Cryonics Institute) claim to have eliminated ice formation in the brain by the use of vitrification solution, but make no such claim for other organs or tissues.^16,17^

Vitrification is solidification to an amorphous (glassy) state, which is distinct from the crystalline state characteristic of ice. Amber is a familiar example of a vitreous (amorphous, non-crystalline) solid. Pure water can be made to vitrify if cooled not more slowly than three million kelvins per second,^18^ a cooling rate impractical for animal tissues. Sucrose can be cooled rapidly enough to be vitrified into “cotton candy,” but with slower cooling, it forms a “rock candy” crystal. Adding corn syrup to sucrose allows it to be cooled slowly to the noncrystalline solid used in lollipops. Silicon dioxide can be rapidly cooled to vitreous silica or can be slowly cooled to the crystalline form (quartz). Common glassware and windowpanes are made by adding sodium and calcium oxides to silicon dioxide to produce a molten liquid that can cool slowly as an increasingly viscous syrup to an amorphous (non-crystalline) solid. In the absence of a phase transition from liquid to solid crystal at melting/fusion temperature, there is a great increase in viscosity (characterized as solidification), which occurs at a glass transition temperature (T_g_) that is determined by cooling rate.

Cryoprotectants most frequently used in cryobiology include dimethylsulfoxide (DMSO) as well as the polyols ethylene glycol (an automobile anti-freeze), propylene glycol (once used to reduce ice crystals in ice cream), and glycerol (used since the 1950s to cryopreserve sperm and blood cells). All of these compounds are capable of hydrogen bonding with water to prevent water molecules from organizing themselves into ice. These cryoprotectants also act by colligative interference that hinders water molecules from forming the ice lattice. Mixtures of cryoprotectants can be less toxic than the pure cryoprotectants and can completely eliminate ice formation. The use of ice blockers (non-cryoprotectant substances such as antifreeze proteins that chemically block ice crystal growth) in vitrification mixtures can further reduce toxicity and concentration needed to vitrify.^19^

Difficulty in achieving sufficiently high cryoprotectant concentration to eliminate ice formation, while at the same time minimizing cryoprotectant toxicity, has been the limiting factor preventing better recovery of biological systems from cryopreservation. Rapid cooling can permit the use of lower cryoprotectant concentrations to prevent ice formation, but rapid cooling becomes increasingly difficult for in-creasingly larger tissues. Cryoprotectant toxicity varies inversely with temperature, so the use of less viscous cryoprotectant mixtures can speed tissue penetration and thereby reduce the tissue cryoprotectant exposure time at higher temperatures before cooling.

A number of possible explanations for cryoprotectant toxicity have been proposed, but the exact molecular mechanisms remain elusive.^20^ Insofar as cryoprotectants do not destroy molecules, the damage they cause may not be irreparable. Moreover, considerable success has been made in reducing the toxicity of vitrification mixtures,^21,22^ and there is no reason to believe that further toxicity reductions cannot be made.

The mammalian organ that has been studied by the greatest number of researchers attempting organ vitrification is the ovary. Variable success has been achieved with the ovaries of a number of species, but the greatest success has been with the mouse ovary. Vitrified mouse ovaries cryopreserved at −196°C have been re-warmed to produce live pup birth rates comparable to that seen with fresh ovaries.^23^

A study conducted with rat hippocampal slices showed that it is possible for vitrified slices cooled to a solid state at −130°C to have viability upon re-warming comparable to that of control slices that had not been vitrified or cryopreserved. Ultrastructure of the CA1 region (the region of the brain most vulnerable to ischemic damage) of the re-warmed slices is seen to be quite well preserved compared to the ultrastructure of control CA1 tissue.^24^ Cryonics organizations perfuse brains with vitrification solution until saturation is achieved.

Tissues that have been vitrified and cryopreserved are assessed for viability as well as for ultrastructure. Intracellular K^+^/Na^+^ ratio is a commonly used method of assessing viability, although other methods (such as measurement of intracellular ATP content) could be useful in the future. The sodium pump, which maintains membrane potential, will not function without binding to ATP and Na^+^ inside the membrane and K^+^ outside the membrane. Although a cell can maintain a membrane potential for several hours without a functional sodium pump, the slow leak of Na^+^ into the cell and consequent leak of K^+^ out of the cell will result in a complete loss of membrane potential after several hours. Similarly, if the cell dies in the sense of no longer being capable of producing energy (ATP) in the mitochondria, the sodium pump will cease to operate. Thus, normal intracellular K^+^/Na^+^ ratios indicate functioning sodium pumps and intact cell membranes.

To assay the intracellular K^+^/Na^+^ ratio, tissues are placed in mannitol to wash away extracellular ions. Trichloroacetic acid is then used to rupture cell membranes and release intracellular ions. A flame photometer or atomic absorption spectrometer can be used to determine the relative concentrations of sodium and potassium ions. Viability studies of vitrified hippocampal slices using intracellular K^+^/Na^+^ ratios indicated viability in excess of 90% normal.^24^

In one study, a rabbit kidney has been vitrified, cooled to −135°C, re-warmed, and transplanted into a live rabbit. The formerly vitrified transplant functioned well enough as the sole kidney to keep the rabbit alive indefinitely.^25^ Some people imagine a need to understand brain function as being essential for brain cryopreservation. But in simple terms, a kidney produces urine and a brain produces consciousness. A re-warmed brain that is physiologically restored should be able to produce consciousness no less than a re-warmed vitrified kidney can produce urine. Preservation of structure and restoration of physiology should result in restoration of function, irrespective of the organ or tissue.

The vitrification mixture used in preserving the rabbit kidney is known as M22. M22 is used by the cryonics organization Alcor for vitrifying cryonics subjects. Perfusion of rabbits with M22 has been shown to preserve brain ultrastructure without ice formation.^26^

Cooling from 0°C to −130°C should be rapid to minimize the possibility of ice formation. When cooling from −130°C to −196°C, thermal stress on large solid vitrified samples can cause cracking and fracturing.^27^ Although it should theoretically be possible to cool to −196°C slowly enough to avoid cracking, the requisite cooling rates are unknown and may be too slow to be practical. Annealing a vitrified sample near glass transition temperature can reduce thermal stress,^28^ but this may not be adequate. Due to its more well-defined nature, cracking damage may be much easier to repair than freezing damage.

## “Reversible Death?”

As recently as the 1950s, it was believed that death is irreversible when the heart stops. Today it is established that cardiopulmonary resuscitation (CPR) in combination with automated external defibrillators (AED) can restore many people to life who were clinically dead because of cardiac arrest.^29^ But it is still widely believed that after about 6 min of cardiac arrest without circulation, irreparable brain damage has already occurred.

In 1976 Peter Safar (the “father of CPR”) showed that dogs could be subjected to 12 min of cardiac arrest without neurological damage by the use of elevated arterial pressure, norepinephrine, heparin, and hemodilution with dextran 40.^30^ Over a decade later, an experiment showed that spontaneous EEG activity returned in 50% of cats subjected to 1 h of global cerebral ischemia followed by reperfusion and treatment with norepinephrine (or dopamine), heparin, insulin, and acidosis buffers. Six out of 15 of the cats submitted to intensive care regained spontaneous respiration, and one of those cats survived a full year with normal neurological function (except slight ataxia).^31^ The 6 min limit is not mainly a neurological phenomenon; it is a problem of increased vascular resistance that can be overcome (in part) by increasing perfusion pressure.^32^

Reperfusion injury refers to the tissue damage inflicted when blood flow is restored after an ischemic period of more than about 20 min. The resupply of blood after an excessive period of ischemia initiates inflammatory processes and causes oxygen to form toxic free radicals (reactive oxygen species) such as superoxide.^33^ Xanthine oxidase-produced superoxide damages the endothelium far more than the parenchyma.^34^ In inflammatory conditions, such as occur in reperfusion, inducible nitric oxide synthetase can increase nitric oxide concentration to thousands of times normal levels.^35^ During reperfusion, abnormally high amounts of superoxide convert almost all available nitric oxide to perxoynitrite, regarded as the agent causing most of the damage to brain capillary endothelial cells.^36^

Despite the damaging effects of excitotoxicity,^37^ brain structure is normally retained post-mortem much longer than is commonly appreciated. In the cerebral cortex of rats subjected to occlusion of blood flow to the cortex (cerebral ischemia), only 15% of neurons were necrotic after 6 h. Most neurons (65%) did not become necrotic until 12 h after the cessation of blood flow.^38^ Neurons isolated from the brains of autopsied elderly humans an average of 2.6 h post-mortem showed 70–90% viability after 2 weeks *in vitro*.^39^

The reason why more than 6 min of cardiac arrest currently leads to neurological damage is in part because the ischemia starts a process of neuron self-destruction (apoptosis) which takes many hours to complete. But therapies are on the horizon that may interfere with apoptosis. Neurons in the CA1 sector of the hippocampus are much more vulnerable to becoming necrotic following ischemia than neurons elsewhere in the brain.^40^ But cell death in the hippocampus following ischemia can be significantly reduced by the use of caspase inhibitors that arrest the apoptotic process.^41^ Caspase inhibitors have also been used to block apoptosis in cryopreserved hematopoietic cells re-warmed from cryogenic temperatures.^42^ Bag-1 protein, which binds pro-apoptotic members of the Bcl-2 protein family, has demonstrated powerful anti-apoptotic effects on rat livers subjected to ischemia/reperfusion injury.^43^

Most neuroscientists agree that the anatomical basis of the mind is encoded in the physical structures of the brain, particularly neuropil connectivity and synaptic strengths^44^ and possibly neuronal epigenetic structure.^45^ The fact that complete absence of electrical activity in the brain does not prevent full neurological recovery^5,46^ supports the proposition that the ultimate basis of consciousness is structural rather than dynamic and can therefore be preserved at cryogenic temperatures.

Significant recovery of cerebral cortex function is possible following stroke, which is associated with redundancy of information storage in the brain.^47–49^ Neural stem cell transplantation therapies have the potential to further augment brain recovery from damage due to ischemia, toxins, and cryopreservation injury.^50^ These considerations increase the amount of damage that may be tolerable for restoration of a human cryonically preserved under suboptimal conditions.

Preservation of brain structure and restoration of brain function are essential to cryonics. Other organs and tissues are not as important because artificial organs and tissue regeneration by stem cells should be easily accomplished by future medicine. Appendage regeneration in salamanders is already being used as a guideline for mammalian regenerative medicine.^51^ Biodegradable 3-D braided fibrous scaffolds^52^ can potentially be used for construction of organs, if not whole bodies.

Conservative cryonics strives to minimize damage and minimize reliance on future molecular repair technologies. In many cases cryonics subjects have experienced less than a minute of cardiac arrest before circulation has been restored. Evidence that the neurological basis of the mind is preserved long beyond the 6 min limit gives hope that molecular medicine to reverse apoptosis and repair damaged blood vessels may allow for recovery of cryonics subjects who did not benefit from prompt treatment. It is unlikely that cryonics is worthless after 6 min without blood circulation. Many tissues are alive when the heart stops, and take hours to die.

Under the best circumstances, cryonics subjects experience virtually no ice formation in the brain. Repairs to vitrified brain tissue that had experienced little ischemic damage could be performed above cryogenic temperatures, along with curing diseases and rejuvenation.

Although there are sound legal requirements for drawing a distinct line between life and death, biological and psychological realities point to a continuum rather than discrete binary states. Consciousness emerges gradually from embryo to fetus to child to adult, and consciousness can diminish gradually in neurodegenerative diseases. After the heart stops, the anatomical basis of the mind (the brain) decomposes over a period of hours and days at a temperature-dependent rate. The non-binary character of consciousness would also become evident in revived cryonics subjects whose brains had been partially destroyed and then repaired, resulting in partial amnesia and partial restoration of original identity.

## Cryonics Procedures

Pretreatment of terminal cryonics subjects to reduce ischemia/reperfusion injury is advisable, but is too rarely done in practice. For example, intravenous injection of the alpha-tocopherol form of Vitamin E (20 mg/kg) 30 min prior to ischemia has been shown to significantly reduce lipid peroxidation and neurological damage.^53^ It is better to include both alpha-tocopherol and gamma-tocopherol because gamma-tocopherol removes peroxynitrite whereas alpha-tocopherol does not.^54^ Vitamin E pretreatment for cryonics patients has the additional advantage of reducing blood clotting and does not have the risk of gastric bleeding associated with aspirin. Many fish oils (especially salmon oil) afford the same benefit, in addition to reducing the risk of cardiac arrest.^55^ Reduced clotting in a cryonics patient is usually a great benefit. But for patients undergoing surgery, Vitamin E and fish oils may be prohibited because of the danger of excessive bleeding.

Cryonics procedures are generally only practiced on subjects who have made contractual and funding arrangements in advance with a cryonics organization (such as Alcor Life Extension Foundation, the American Cryonics Society, or the Cryonics Institute). In optimal circumstances, a cryonics subject will be pronounced legally dead very quickly after their heart stops. Only after legal death has been pronounced can the cryonics procedures begin.

Once cardiac arrest has occurred and death has been pronounced, a cryonics subject can be given medications to maintain sedation, reduce cerebral metabolism, prevent/reverse blood clotting, increase blood pressure, stabilize pH against acidosis, and protect against ischemia/ reperfusion injury.

Cryonics procedures involve restoring blood circulation and respiration as soon as possible to keep tissues alive. In cryonics, this is called cardiopulmonary support (CPS) rather than cardiopulmonary resuscitation (CPR) because resuscitation after death has been pronounced is not desired (a do not resuscitate [DNR] condition). Propofol (2,6-diisopropylphenol) is given in part because its sedative action can prevent resuscitation, with the added benefit that it can be neuroprotective.^56^ Propofol has been shown to inhibit the neural cell apoptosis that can occur as a consequence of ischemia/reperfusion injury.^57^

Heparin is used to prevent blood clotting. Streptokinase is the usual thrombolytic used to break up blood clots. THAM (tris-hydroxymethyl aminomethane) is a buffer that maintains arterial pH without producing carbon dioxide and also maintains intracellular pH because it readily crosses cell membranes.^58^

When the equipment is available, cryonics teams restore circulation and respiration with mechanical devices capable of restoring circulation on the down-stroke (compression) as well as the up-stroke (decompression). Active compression-decompression (ACDC) and interposed abdominal compression can improve CPS perfusion considerably.^59^ Epinephrine has commonly been used to supplement CPS by maintaining blood pressure, although vasopressin may also be used.^60^

While the cryonics subject is receiving ACDC CPS, he or she is in a bath of circulating ice water. Cooling is much more rapid in water than in air^61^ and is much more rapid in flowing water than in still water, according to Newton's law of cooling. The cooling of the cryonics subject is also considerably hastened by the blood circulation resulting from ACDC CPS.

Once the cryonics subject is cooled to below 10°C, perfusion with vitrification solution can begin. Vitrification of the brain is achieved by cryoprotectant exposure times, which are considerably longer than those seen for vitrification of hippocampal slices. Cryoprotectants are toxic, so increased cryoprotectant exposure time means increased toxicity to the exposed tissues. But large organs and body tissues cannot be cooled as rapidly as tissue slices, therefore higher cryoprotectant concentrations must be used to prevent ice formation.

Suggestions have been made for systems to store cryonics subjects at temperatures closer to −130°C to eliminate cracking due to thermal stress in cooling to −196°C. Subjects stored just below −130°C would still be in the solid state, insofar as vitrification solutions have a glass transition temperature just below −120°C.^20^ Storage just below −130°C has yet to be implemented on all but a few cryonics subjects.

Cryonics subjects are stored in thermos bottle-like containers of liquid nitrogen, a storage method that is both inexpensive and not dependent upon electricity (and thus, not so vulnerable to power failure).

## Science and Indirect Evidence

Many critics assert that cryonics is not science and has no capability of becoming science until a mammal has been re-warmed and reanimated after having been cryopreserved at cryogenic temperatures. However, as demonstrated through scientific history, model building based on extrapolations from indirect evidence is central to science.

No one has ever seen the core of the Earth. Models have been constructed of the state of the universe in the first millionth of a second following the Big Bang. Scientists describe the state of the Earth after years of global warming. Models have been constructed of the state of contained nuclear waste hundreds of thousands of years in the future, models upon which considerable reliance is placed in the disposal of nuclear waste. Past landings by humans on the moon provide indirect evidence that future landings of humans on Mars may be possible.

Many people cryopreserve umbilical cord stem cells from their newborn baby based on the future potential of scientific developments rather than on the basis of current science. Germ cells and DNA from endangered species are similarly cryopreserved in anticipation of future technology. Influenza vaccines only have a 50% chance of protecting a person over 65.^62,63^ It is not unscientific to risk modest or heroic medical treatments that are justified by indirect evidence for some probability of success rather than for absolute guarantee of success.

If there are plausible models for the repair and reanimation of cryopreserved cryonics subjects,^64,65^ it seems reasonable to rely upon them when deciding on human cryopreservation as a long-term treatment that may or may not succeed. To wait until a cryopreserved mammal has been reanimated before cryopreserving humans could mean that many lives will be lost. It would be like waiting tens of hundreds of thousands of years to ensure that nuclear waste can be contained before containing those wastes. Indirect evidence exists to support the claim that revival of a cryopreserved mammal is not essential for cryonics practice to be scientifically justified.

## Conclusions

Low temperatures slow biological time, effectively stopping time at liquid nitrogen temperature. Cryoprotectants greatly reduce damage caused by tissue cryopreservation, and effective vitrifications can prevent ice formation completely. Cryoprotectant toxicity is likely a reparable injury, and less toxic means of cryopreservation continue to be discovered. Applied to humans and animals, cryopreservation is a means of achieving a stable biological state that is reversible in principle.

Dying is a process that begins, not ends, when heartbeat and blood circulation stop. When legal death is declared based on cardiopulmonary arrest, cryonics procedures aim to minimize further injury by artificially restoring blood circulation and rapidly reducing temperature. The duration of clinical death at warm temperatures beyond which the brain information that defines a human being is lost may be many hours.

The proposition that aging is a disease that can be treated and perhaps eventually be reversed (rejuvenation), is based on the general understanding that aging consists of a multitude of specific pathologies on cellular and molecular levels that can be studied, understood, and reversed with foreseeable tools. Pathologies caused by global cerebral ischemia (clinical death), by cryopreservation, and by other presently incurable diseases are similarly amenable to analysis and possible future repair. If aging damage can be repaired at some future time, it is not unreasonable to think that damage due to cryonics procedures can also be repaired. And if aging damage can be repaired at some future time, cryonics may be the only way for many people living today to obtain future medical procedures that can cure presently incurable diseases and thereby rejuvenate.
